# Functional protease profiling with reporter peptides in serum specimens of colorectal cancer patients: demonstration of its routine diagnostic applicability

**DOI:** 10.1186/1756-9966-31-56

**Published:** 2012-06-08

**Authors:** Peter Findeisen, Victor Costina, Diego Yepes, Ralf Hofheinz, Michael Neumaier

**Affiliations:** 1 Institute for Clinical Chemistry, Medical Faculty Mannheim of the University of Heidelberg, University Hospital Mannheim, Theodor-Kutzer-Ufer 1-3, Mannheim, 68167, Germany; 2 III. Medical Clinic, Medical Faculty Mannheim of the University of Heidelberg, University Hospital Mannheim, Theodor-Kutzer-Ufer 1-3, Mannheim, D - 68167, Germany

**Keywords:** Functional protease profiling, Serum, Colorectal cancer, Cancer procoagulant diagnosis, Reporter peptide, Mass spectrometry

## Abstract

**Background:**

The progression of many solid tumors is characterized by the release of tumor-associated proteases and the detection of tumor specific proteolytic activity in serum specimens is a promising diagnostic tool in oncology. Here we describe a mass spectrometry-based functional proteomic profiling approach that tracks the *ex-vivo* degradation of a synthetic endoprotease substrate in serum specimens of colorectal tumor patients.

**Methods:**

A reporter peptide (RP) with the amino acid sequence WKPYDAAD was synthesized that has a known cleavage site for the cysteine-endopeptidase cancer procoagulant (EC 3.4.22.26). The RP was added to serum specimens from colorectal cancer patients (n = 30), inflammatory controls (n = 30) and healthy controls (n = 30) and incubated under strictly standardized conditions. The proteolytic fragment of the RP was quantified with liquid chromatography / mass spectrometry (LC/MS).

**Results:**

RP-spiking showed good intra- and inter-day reproducibility with coefficients of variation (CVs) that did not exceed a value of 10%. The calibration curve for the anchor peptide was linear in the concentration range of 0.4 – 50 μmol/L. The median concentration of the RP-fragment in serum specimens from tumor patients (TU: 17.6 μmol/L, SD 9.0) was significantly higher when compared to non-malignant inflammatory controls (IC: 11.1 μmol/L, SD 6.1) and healthy controls (HC: 10.3 μmol/L, SD 3.1). Highest area under receiver operating characteristic (AUROC) values were seen for discrimination of TU versus HC (0.89) followed by TU versus IC (0.77). IC and HC could barely be separated indicated by an AUROC value of 0.57. The proteolytic activity towards the RP was conserved in serum specimens that were kept at room temperature for up to 24 hours prior to the analysis.

**Conclusion:**

The proteolytic cleavage of reporter peptides is a surrogate marker for tumor associated proteolytic activity in serum specimens of cancer patients. A simple, robust and highly reproducible LC/MS method has been developed that allows the quantification of proteolytic fragments in serum specimens. The preanalytical impact of sample handling is minimal as the tumor-associated proteolytic activity towards the reporter peptide is stable for at least up to 24 h. Taken together, the functional protease profiling shows characteristics that are in line with routinely performed diagnostic assays. Further work will focus on the identification of additional reporter peptides for the construction of a multiplex assay to increase diagnostic accuracy of the functional protease profiling.

## Background

Proteases play an important role in different biological processes including cell differentiation, inflammation and tissue remodelling, haemostasis, immunity, angiogenesis, apoptosis and malignant disease [1]. Specifically, proteases are well known factors to promote local progression and distant metastasis of colorectal cancer and many other solid tumors [2,3]. Furthermore, there is increasing evidence that proteases also have key functions in early stages of tumor development [4]. The tumor-associated proteases are either secreted directly by the tumor or originate from surrounding connective tissue and infiltrating leucocytes as a result of tumor-stroma interaction [5]. Some tumor-associated proteases like cathepsins, matrix-metalloproteases, kallikreins and cancer procoagulant (CP) are released into the bloodstream and can be used for diagnostic and prognostic purposes [6-10]. Tumor-associated protease activity in serum specimens of cancer patients can be monitored using synthetic substrates that are selectively cleaved by the protease of interest [6-9]. With the use of appropriate synthetic reporter-peptides (RPs) for spiking of serum specimens, the reaction conditions that comprise substrate concentration, incubation time and buffer composition can be optimized and standardized accordingly [11]. Furthermore, the proteolytic fragments accumulate to the level that they become readily detectable by mass spectrometry [8]. This approach is similar to established diagnostic assays measuring the proteolytic activity of distinct enzymes, e.g., coagulation factors [12].

Recently, we have described a functional protease profiling approach using a reporter peptide that is cleaved by the tumor associated protease cancer procoagulant (EC 3.4.22.26) [8]. However, the analysis of proteolytic fragments was performed with MALDI-TOF mass spectrometry that is only a semi-quantitative method [13] with limited inter-day reproducibility [8]. Furthermore, proteolytic fragments had to be extracted from serum specimens with serial affinity purification that is a rather laborious method with limited throughput and reproducibility. To alleviate these restrictions, we have developed a robust and highly reproducible liquid chromatography-mass spectrometry (LC-MS) assay for the absolute quantification of a targeted proteolytic fragment.

Serum has a high intrinsic proteolytic activity that leads to continuous processing of proteins and peptides [14]. To protect the reporter peptide from unwanted and unspecific processing by exopeptidases the cleavage site WKPYDAAD is flanked by aminohexanoic acid (see Table 1). When the reporter peptide is cleaved by the endoprotease cancer procoagulant after the tyrosine (Y) [15], the resulting free amino-terminus of the intermediate fragment is rapidly trimmed down by aminopeptidases [8]. This results in the accumulation of a protease resistant anchorpeptide (CP-AP) that consists of aminohexanoic acid and D-aminoacids (see Table 1). The anchorpeptide was quantified by liquid chromatography / mass spectrometry (LC/MS) with good reproducibility that is in line with routinely performed diagnostic tests.

**Table 1:** Peptide sequences of reporter peptide, anchor peptide and internal standard

**Name**	**Peptide sequence**	**[M + H]**^**2+**^**observed**	**[M + H]**^**1+**^**theoretical (monoisotopic)**
CP-RP	Ahx-WKPYDAAD-Ahx-ateeqlkv		2.090,06
CP-AP	Ahx-ateeqlkv	515,795	1.030,59
IS	Ahx-ateevlkl	508,300	1.015,61

CP-RP: Cancer Procoagulant-Reporter Peptide. CP-AP: Cancer Procoagulant-Anchor Peptide. IS: Internal Standard. Ahx: amino hexanoic acid. Lower case letters indicate D-amino acids.

The sufficient preanalytical stability of biomarkers is a prerequisite for routine diagnostic use and we could demonstrate that the tumor-associated proteolytic activity towards the reporter peptide is preserved for up to 24 h. Furthermore a small proof-of-concept experiment (n = 90) was performed to demonstrate the diagnostic power of functional protease profiling with reporter peptide spiking. Systemic inflammation has been recognized as serious threat for cancer biomarker discovery [16] and we selected the collective of control individuals accordingly. The concentrations of proteolytic fragments were significantly higher in serum specimens from tumor patients (TU) when compared to serum from inflammatory controls (IC) and healthy controls (HC). This indicates the presence of the tumor-associated protease cancer procoagulant that is associated with an increased cleavage of the reporter peptide in serum specimens of tumor patients.

Here we present a method to monitor controlled, *ex-vivo* peptide breakdown in serum samples using LC/MS with absolute quantification of the respective fragment that might lead to an activity based approach for biomarker discovery and validation.

## Results

### LC-MS analysis and absolute quantification of the anchor peptide

**Figure 1:**
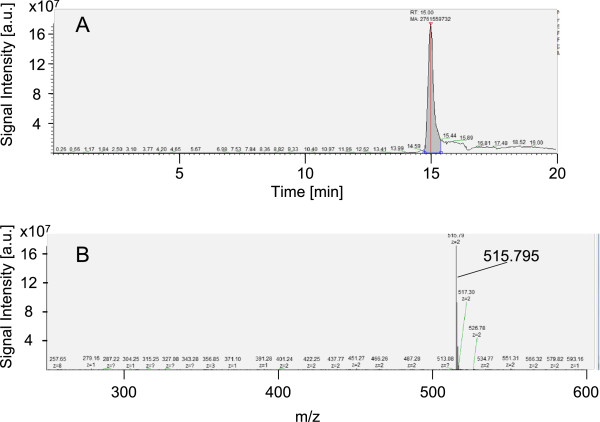
**Exemplary LC/MS results.** LC/MS results of the calibration standard with CP-AP concentration of 0.4 μmol/L (**A**) Extracted ion chromatogram (XIC) of CP-AP with extracted mass of 515.795 +/−0.005. The peak area of the respective m/z 515.795 is filled in grey and was used for quantification. (**B**) ESI mass spectrum of the anchor peptide eluting at 15 +/− 1 min.

**Figure 2:**
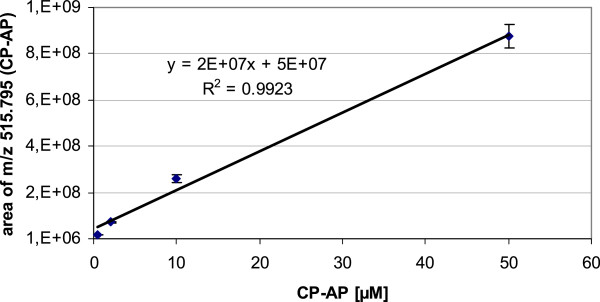
**Calibration curve of anchor peptide m/z 515,795.** Measurements for each CP-AP concentration (0.4; 4; 20 and 50 μmol/L) were performed in triplicate and linear regression was calculated with median values. Error bars indicate the standard deviation. Coefficient of determination (R^2^) is displayed in the graph.

The proteolytic cleavage of the reporter peptide (CP-RP) by the endoprotease cancer procoagulant results in an accumulation of the anchor peptide (CP-AP). The amino acid sequence WKPYDAAD of CP-RP is specifically cleaved after the aminoacid tyrosine (Y) by the endoprotease cancer procoagulant prior to further processing by serum exopeptidases [8,15]. Finally, the protease-resistant anchor peptide (CP-AP) m/z 515.795 which consists of the linker and D-amino acids (Table 1) is accumulating and high concentration is a surrogate marker for increased proteolytic activity of cancer procoagulant. Figure 1 gives a representative example of the LC/MS results for CP-AP. Figure 1A shows the extracted ion chromatogram (XIC) of CP-AP and labelling of the respective peak area that was used for quantification. Figure 1B shows the corresponding mass spectrum within the selected mass window ranging from m/z 250 to m/z 600. Note that only one peak with the respective isotopic pattern exceeded the signal intensity of 2 × 10^7^ [a.u.]. This m/z 515.795 was expected to be the doubly charged molecule CP-AP (Table 1) and the sequence was verified by tandem mass spectrometry (Additional file 1: Figure S1). The mass spectra of the internal standard (IS) are of equal quality regarding the signal to noise ratio (data not shown). A calibration curve was prepared using pooled serum of healthy controls that was spiked with four different concentrations of CP-AP ranging from 0.4 to 50 μmol/L. The linearity of the calibration curve within this concentration range was good with a coefficient of determination (R^2^) of 0.992 (Figure 2).

### Optimization of incubation time and reproducibility of RP-spiking

**Figure 3:**
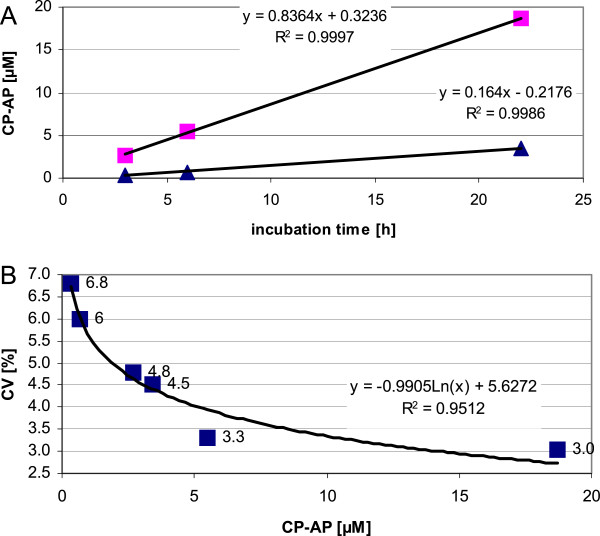
**Kinetic measurements of CP-AP in pooled serum specimens of tumor patients and healthy controls.** (**A**) Accumulation of CP-AP correlates with incubation time. Linear regression was calculated from median values of five measurements. Squares: pooled serum specimen from tumor patients. Triangles: pooled serum specimen from healthy controls. The equations of the linear regression and coefficients of determination (R^2^) are displayed in the graph. (**B**) Intraday-reproducibility. Inverse correlation of concentrations of CP-AP and coefficients of variations (CVs) for five repetitive measurements. The CVs (y-values) are shown next to the squares in the graph. A logarithmic regression has been calculated with Excel (Microsoft) and the equation and coefficients of determination (R^2^) are also displayed in the graph.

The quantification of the anchor peptide CP-AP is performed as mass-spectrometric endpoint-assay and the appropriate incubation time has to be determined. As expected, the concentration of CP-AP is constantly increasing during prolongation of the incubation time from 3 h to 6 h and 22 h (Figure 3A). The accumulation of CP-AP is approximately five times faster in the tumor serum (QCT), when compared to a healthy control specimen (QCH) as indicated by the linear regression graphs with slopes of 0.836 and 0.164 respectively (Figure 3A). The incubation for 22 h seems to be preferable as reproducibility of measurements is improved with increasing signal intensity that is associated with prolonged incubation time [17]. The CVs are inversely correlated to the signal intensity and range from 6.8% to 3.0% for CP-AP concentrations of 0.33 μmol/L and 18.7 μmol/L respectively (Figure 3B). Consequently, an incubation period of 22 h was chosen for any further experiments.

### Inhibition of proteolytic reaction with iodoacetamide

The cysteine-endoprotease cancer procoagulant can specifically be inhibited by iodoacetamide [18] and different concentrations of protease inhibitor were added to spiked serum specimens of a tumor patient. As expected, the concentration of CP-AP is inversely proportional to the amount of iodoacetamide concentrations of serum specimens that were spiked with CP-RP. After 22 h of incubation the amount of CP-AP that accumulated in the serum specimen was taken as 100%. In the presence of 5, 25 and 100 mmol/L jodoacetamide, the CP-AP concentration was reduced down to 88%, 63% and 25% respectively (Additional file 2: Figure S2).

### Preanalytical stability of cancer procoagulant activity

**Figure 4:**
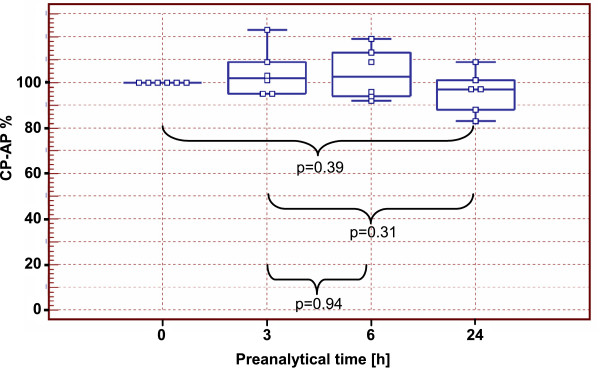
**Preservation of protease activity in a preanalytical time period of 24 h.** Aliquots of serum specimens from 6 tumor patients were frozen at −80°C directly after centrifugation (0 h) or after prolonged preanalytical time span of 3 h, 6 h, and 24 h. After thawing, specimens were spiked with CP-RP and incubated for 22 h prior to peptide extraction with TCA and LC-MS. CP-AP peak areas were extracted from the data. The CP-AP concentrations of the freshly obtained serum aliquots (0 h) were set to 100%. In the box plot the central box represents the values from the lower to upper quartile (25 to 75 percentile). The middle line represents the median. The horizontal line extends from the minimum to the maximum value. P-values of the Mann–Whitney test are indicated.

Serum specimens from 6 tumor patients were aliquoted and stored 0, 3, 6 and 24 h at room temperature prior to freezing at −80°C. After thawing, reporter peptide CP-RP was added to serum specimens and incubated 22 h under standardized conditions as described in materials and methods. The concentrations of CP-AP in the serum specimens without preanalytical time delay (0 h) ranged from 4.27 μmol/L to 13.14 μmol/L and were set to 100%. Compared to freshly prepared specimens (0 h) the CP-AP concentrations after 3, 6 and 24 h of preanalytical time had median values of 103%, 102% and 97% respectively (Figure 4). The concentrations of CP-AP in serum specimens with prolonged preanalytical time span (3 h, 6 h, 24 h) were not significantly different from concentrations that were measured in fresh specimens (0 h). This indicates that cancer procoagulant activity towards the reporter peptide is stable at least over a preanalytical time period of 24 h.

### Functional protease profiling

Finally, we set up a proof-of-concept experiment to elucidate the applicability of protease profiling for diagnostic purposes. However, the implementation of MS as a routine diagnostic tool clearly depends on good inter-day reproducibility of the method. Three aliquots of a serum specimen from one tumor patient were randomly integrated into small series of serum specimens from patients and control individuals on four consecutive days. The median concentration of CP-AP was 31.9 μmol/L with SD of 3.3 μmol/L and CV of 10.2% (Additional file 3: Figure S3). As expected, the inter-day reproducibility is not as good as the intra-day reproducibility (see Figure 3B). However, CVs of 10% or even more are acceptable for many routine laboratory assays [19].

**Figure 5:**
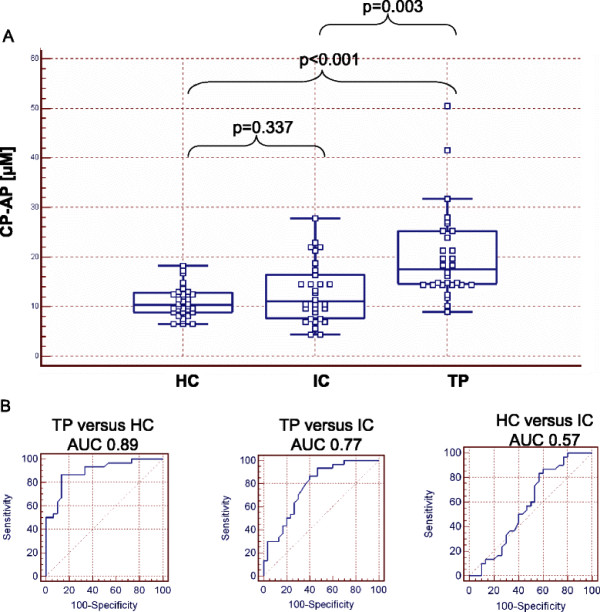
**Proof-of-concept experiment for functional protease profiling with reporter peptide spiking.** Serum specimens were spiked with CP-RP and IS and incubated for 22 h as described in materials and methods. (**A**) CP-AP concentrations in serum specimens of healthy controls (HC), inflammatory controls (IC) and tumor patients (TP). In the box plot the central box represents the values from the lower to upper quartile (25 to 75 percentile). The middle line represents the median. The horizontal line extends from the minimum to the maximum value. P-values of the Mann–Whitney test are indicated. (**B**) ROC-AUC calculation for separation of tumor patients (TP) from healthy controls (HC) (left graph), tumor patients (TP) from inflammatory controls (IC) (middle graph) and healthy controls from inflammatory controls (IC) (right graph).

Serum specimens from patients with metastatic colorectal tumors (TP = 30), patients without malignant disease but elevated acute phase protein CRP (IC = 30) and healthy controls (HC = 30) were spiked with CP-RP and internal standard (IS). Samples were incubation for 22 h and sample preparation prior to LC-MS was performed as described in materials and methods. The median concentrations of CP-AP in the collectives of healthy controls (HC), inflammatory controls (IC) and tumor patients (TP) were 10.3 (SD 3.1), 11.1 (SD 6.1) and 17.6 (SD 9.0) respectively (Figure 5A). The D’Agostino-Pearson test was used to asses the normal distribution within the reporter peptide concentrations. For HC and IC the p-values were higher than 0.05 indicating a normal distribution. However, for TU the p-value was <0.05 and the hypothesis that the distribution of the observations in the sample is normal, was rejected. Accordingly, further data analysis was performed with the non-parametric Mann–Whitney test. The concentrations of CP-AP were not significantly different, when HC versus IC was compared with the Mann–Whitney test (p = 0.337). In contrast, the comparison of HC versus TP and IC versus TP showed statistically significant differences with p values below 0.005 (Figure 5A). The diagnostic accuracy for discrimination of healthy controls and tumor patients was calculated with receiver operating characteristics (ROC) that had an area under the curve (AUC) of 0.89. The ROC-AUC for discrimination of inflammatory controls and tumor patients had a value of 0.77. The 95% confidence intervals ranged from 0.787 to 0.958 and from 0.646 to 0.871 respectively. In contrast, inflammatory controls and healthy controls could not be differentiated with a ROC-AUC of 0.57 with 95% confidence interval ranging from 0,438 to 0,699 (Figure 5B). These data suggest that the activity of the tumor-associated endoprotease cancer procoagulant is increased in serum specimens of tumor patients when compared to healthy and inflammatory controls.

## Discussion

The dysregulation of protease activity plays an important role for the initiation and progression of malignant disease [1,4]. Tumor-associated proteases like matrix metalloproteases, cathepsins, kallikrein related peptidases and members of the plasminogen activator system are secreted into the bloodstream and might be candidates for functional protease profiling (for review see [20]). Specifically, the tumor-associated protease cancer procoagulant is secreted from numerous malignancies including colorectal cancer into the bloodstream [21]. Under *in vivo* conditions this can cause paraneoplastic coagulopathy throughout cleavage and activation of the coagulation factor X heavy chain (P00742) [22]. The reporter peptide CP-RP comprises the cleavage site WKPYDAAD that is part of the coagulation factor X and is preferably cleaved in serum specimens of tumor patients [8]. Adding reporter peptides to serum specimens enables the monitoring of tumor-related proteolytic activity for diagnostic use [7-9,23,24]. Furthermore, reporter peptide spiking offers major advantages over native MS-based peptide profiling concerning the standardization of preanalytical variabilities [6,11]. The main focus of our present work was to optimize functional protease profiling with respect to simplified sample preparation and increased inter-day reproducibility to make it amenable as a laboratory assay for routine diagnostic use.

Recently, a sample clean-up with trichloroacetic acid (TCA) has been described that showed a sufficient recovery for peptides with a molecular weight of less than 3000 Da [25]. Furthermore, the LC-MS technique is the method of choice for the reproducible quantification of small molecules like peptides in clinical specimens [26], and accordingly this technology was selected for assay development. Even at low CP-AP concentrations of 0.4 μmol/L the extracted ion chromatogram of CP-AP with m/z 515.795 shows only one single peak (see Figure 1) and this excellent signal to noise ratio makes quantitative LC/MS analyses amenable [27,28]. Recently, criticism has been raised against functional protease profiling and it has been suggested to characterize the proteolytic activity in more detail [29]. Here, we demonstrate that the proteolytic processing of CP-RP and thus the accumulation of CP-AP can be inhibited by the addition of a protease inhibitor. Iodoacetamide is a known cysteine protease inhibitor and reacts readily with the free thiol of cysteine residues required for the hydrolyzing proteases such as cancer procoagulant [18,30]. The amount of CP-AP that is generated in the serum of cancer patients is inversely proportional to the concentration of iodoacetamide added (Additional file 2: Figure S2). This demonstrates that the cleavage of CP-RP and the accumulation of CP-AP is a specific reaction that is related to cysteinprotease activity.

Most interestingly, the proteolytic activity of serum specimens towards CP-RP is conserved for up to 24 h indicating a good preanalytical stability making it useful for diagnostic application (Figure 4).

One major challenge of functional protease profiling is the appropriate selection of exogenous reporter peptides, which are exclusively cleaved by tumor-associated proteases. However, serum is a difficult matrix with high intrinsic proteolytic activity caused by different endoproteases e.g. from the coagulation cascade and the complement system [14,31,32] as well as a multitude of exoproteases [33]. Furthermore, the proteolytic profile in blood specimens is not only altered in malignant disease but also under non-malignant conditions e.g. inflammation [16]. In order to be useful for diagnostics, such proteolytic patterns must be distinguishable from e.g. the inflammatory responses in unrelated non-malignant conditions. As these patterns overlap to a great extent, the classification of tumour patients on the basis of proteolytic activity is a demanding task. Our study addresses this important question by demonstrating the diagnostic accuracy of functional protease profiling with exogenous reporter peptides in a proof-of-concept experiment including patients with inflammatory conditions during non-malignant diseases into the control cohort. Most importantly, there were no statistically significant differences of CP-AP concentrations between the healthy controls and inflammatory controls, while CP-AP concentrations were significantly higher in serum specimens from tumor patients (see Figure 5A). This indicates that changes of the proteolytic profile related to inflammation do not affect the specific processing of the reporter peptide CP-RP. However, we emphasize that this small proof-of-principle profiling experiment has serious shortcomings concerning the limited number of analyzed specimens and the selection of late-stage tumor patients with highly elevated CEA concentrations (see Table 2). Further studies will have to integrate also early tumor stages and in addition should evaluate the impact of therapeutic interventions to clarify the potential benefit of functional protease profiling. Finally, it is likely that tumor heterogeneity during progression of malignant disease may result in different protease patterns [34]. Based on the use of one single reporter peptide as shown here this needs to be accommodated to ensure high diagnostic accuracy and accordingly the areas under the ROC curves were ‘only’ 0.89 and 0.77 for the discrimination of tumor patients versus healthy controls and tumor patients versus inflammatory controls respectively (see Figure 5B). To increase the diagnostic accuracy of functional protease profiling, it seems reasonable to combine different reporter peptides for multiplex analysis that has potentially superior diagnostic accuracy [35]. To achieve this goal, it will be necessary to systematically identify reporter peptide sequences that are most efficiently cleaved by disease-specific proteases. However, any multiplex assay for functional protease profiling might implement the development of kinetic measurements and the need for chromogenic protease substrates [36]. Further work will focus on the identification of additional reporter peptides that are cleaved by other tumor-associated proteases e.g. metalloproteases, cathepsins or kallikreins in order to construct a multiplex protease profiling assay with increased diagnostic sensitivity and specificity.

**Table 2:** Patient demographics and clinical characteristics

	**Diagnosis**		**CEA [μg/l]**		**CRP [mg/l]**		**Sex**		**Age**	
**Classification**	**Disease**	**n**	**Mean**	**SD**	**Mean**	**SD**	**Male**	**Female**	**Mean**	**SD**
HC	not reported	30	3,3	1,3	3,3	2	10	20	50,0	9,4
IC	tissue damage	13	2,8	1,4	146,9	61	19	11	68,9	12,2
	pneumonia	7								
	UTI	4								
	IBD	2								
	pancreatitis	2								
	sepsis	2								
TU	CRC	30	597,6	1014,7	10,9	7	14	16	66,2	10,4

HC; healthy controls. IC; inflammatory controls. TU; tumor patients. UTI; urinary tract infection. IBD; inflammatory bowel disease. Reference range of CEA: <5 μg/l. Reference range of CRP: <5 mg/l.

## Conclusion

Here we present an optimized LC/MS assay for the quantification of a reporter peptide fragment that correlates with tumor-associated proteolytic activity in serum specimens of colorectal cancer patients. With this improved method three major observations could be made: First, the reproducibility of the assay is excellent with coefficients of variation that did not exceed 10%. Second, the tumor-associated proteolytic activity towards the reporter peptide is stable in serum specimens for up to 24 hours. Specifically, good reproducibility and sufficient preanalytical stability are major prerequisites of laboratory diagnostic assays. Third, inflammatory controls (IC) could fairly be separated from tumorpatients (TP) and this is most important as inflammation is an inherent component of cancer and many studies have identified biomarkers that are associated with inflammation rather than malignancy [16]. However, there is a considerable overlap concerning the concentration of CP-AP in serum specimens from controls and tumorpatients. The combination of multiple reporter peptides that are processed by different tumor-associated proteases will be necessary to increase diagnostic accuracy of functional protease profiling. However, if suitable reporter peptides are available, the simultaneous quantification of multiple anchorpeptides could easily be adopted for the presented LC/MS method.

## Materials and methods

### Materials and chemicals

The reporter peptide (CP-RP), the anchor peptide (CP-AP) and the internal standard (IS) (Table 1) were synthesized in the functional genome analysis laboratory of the German Cancer Research Centre (Heidelberg, Germany). HPLC-grade acetonitrile was purchased from Fisher Chemicals (Germany). Formic acid was purchased from Sigma (Germany). Phosphate buffered saline pH 7.4 (PBS) was purchased from PAA Laboratories. Protease buffer: 200 mol/L TrisHCl, 20 mmol/L CaCl_2_, pH 7.8. Iodoacetamide and trichloroacetic acid were purchased from Sigma and Fluka respectively. All reagents and chemicals were at least of analytical grade.

### Serum samples

Whole blood specimens were acquired from patients with metastatic colorectal tumors (n = 30) and patients without malignant disease but elevated acute phase protein CRP (n = 30) at the University Hospital Mannheim. Blood from healthy control individuals (n = 30) was taken from employees of the University Hospital Mannheim during routine laboratory testing at the works doctor’s office. Patient characteristics are summarized in Table 2. Blood collection was performed after we obtained institutional review board approval and patients’ written informed consent. After a 30 min clotting time at room temperature the specimens were centrifuged at 20°C for 10 min at 3000 x g. The serum was aliquoted and stored at −80°C until further use. All serum specimens were refrigerated within 6 hours after blood withdrawal. Any handling and processing of serum specimens from tumor patients and controls was performed in a strictly randomized and blinded manner. Measurements of C-reactive protein (CRP) and carcinoembryonic antigene (CEA) were performed on the Dimension Vista^TM^ System (Siemens).

### Sample preparation

Serum specimens were diluted in the ratio of 1:3 with PBS to a final volume of 100 μL. The reporter peptide (CP-RP) and the internal standard (IS) were dissolved in protease buffer to a concentration of 100 μmol/L for CP-RP and 20 μmol/L for the IS. The diluted serum (50 μL) and the mix of RP and IS (50 μL) were incubated at 37°C for 3 h, 6 h or 22 h as depicted in results. The incubation was terminated by adding 100 μL of 10% (v/v) trichloroacetic acid (TCA) and the resulting mixture was kept at 4°C for 30 min prior to centrifugation for 15 min. at 4°C and 12.000 rpm in a microcentrifuge (Eppendorf). The supernatant was again centrifuged for 5 min. at 4°C and 12.000 rpm and 2 μL of the supernatant were injected onto the HPLC-column.

### Liquid chromatography – mass spectrometry (LC-MS) analysis

LC-MS was performed using a nano HPLC system (UltiMate3000, Dionex) coupled to a linear ion trap Fourier Transform Ion Cyclotron Resonance mass spectrometer (LTQ-FTICR, Thermo Fisher Scientific) with a chip interface (TriVersa NanoMate, Advion). Analytical chromatography of CP-AP and IS (see Table 1) was performed on a 75 μm ID C-18 column (Dionex) with a flow of 300 nL/min and a gradient from 20-35% of buffer B in 23 min. The composition of buffer A was water with 0.1% formic acid and buffer B was 80% acetonitrile with 0,08% formic acid. Each LC run was preceded by a blank run ensuring lack of carryover of the material from the previous run. MS analysis was performed in positive ion mode, with a mass range of 250–600 m/z. MS/MS analyses were performed on the reporter peptide fragment CP-AP for sequence confirmation.

### Reproducibility of reporter peptide spiking

To monitor the reproducibility of reporter peptide spiking, two distinct quality control samples were generated comprising serum specimens from five colorectal tumor patients (QCT) and five healthy control individuals (QCH), respectively. Both samples were aliquoted and stored at −80°C until further use. The QCT and QCH-samples were spiked with the reporter peptide and internal standard and incubated for 3 h, 6 h and 22 h at 37°C as described above. The proteolytic processing of the reporter peptide CP-RP resulted in the accumulation of CP-AP and the respective peak areas were used for quantification using LCQuan that is part of the Xcalibur software package (Thermo Fisher Scientific). Each QC-specimen was processed 5 times and median, standard deviation (SD) and coefficient of variation (CV) of the m/z 515.795 peak was calculated with Microsoft Excel software.

### Statistics

The D’Agostino-Pearson test, Mann–Whitney test and the receiver operating characteristics (ROC) calculations were performed with MedCalc (MedCalc Software). Results for continuous variables were expressed with the medians and standard deviations. Calculated P values of less than 0.05 were considered to indicate statistical significance. Correlation analyses were performed with Microsoft Excel 2002 SP-2 using the ‘add trendline’ functionality.

## Abbreviations

RP: Reporter Peptide; CRP: C-reactive protein; LC/MS: Liquid chromatography mass spectrometry; CV: Coefficient of variation; SD: Standard deviation; CP: Cancer procoagulant; MALDI-TOF-MS: Matrix-assisted laser desorption/ionization–time of flight mass spectrometry; TP: Tumor patients; IC: Inflammatory controls; HC: Healthy controls; CP-RP: Reporter peptide; CP-AP: Anchor peptide; IS: Internal standard; HPLC: High pressure liquid chromatography; m/z: Mass to charge ratio; QC: Quality control; QCT: Pooled serum specimens from tumor patients designated as ‘quality control tumor’; QCH: Pooled serum specimens from healthy controls designated as ‘quality control healthy’; ROC: Receiver operating characteristic; AUC: Area under the curve; XIC: Extracted ion chromatogram; Ahx: Aminohexanoic acid; Abu: 2-aminobutyric acid; a.u.: Arbitrary units.

## Competing interests

The authors declare that they have no competing interests.

## Authors’ contributions

PF planned the experiments and wrote the manuscript, VC and DY performed the mass spectrometric measurements and the data analyses. RH was responsible for the design of the study and MN participated in the manuscript preparation and revised it critically. All authors read and approved the final manuscript.

## Supplementary Material

Additional file 1 **Figure S1:** Amino acid sequence confirmation of the anchor peptide Ahx-ateeqlkv (see Table 1). Print screen of the MS/MS spectra decoding of m/z 515.795 that was performed with PEAKS software (Bioinformatics Solutions). The unusual amino acid Ahx cannot be handled by the software and instead is displayed as Lysine (L) that is an isomer of Ahx and thus produces a fragment with the same mass. Click here for file

Additional file 2 **Figure S2:** Inhibition of protease-activity with iodoacetamide. The protease inhibitor iodoacetamide together with CP-RP and IS was added to a serum specimen from one tumor patient and incubated for 22 h prior to LC-MS analyis. Iodoacetamide concentrations ranged from 5 to 25 and 100 mmol/L. The CP-AP concentration of the serum specimen without iodoacetamide was set to 100%. Measurements were performed in triplicate and the squares indicate median values, error bars indicate standard deviations. The exponential regression was calculated with Excel (Microsoft) and the coefficient of determination (R^2^) is shown in the graph. Click here for file

Additional file 3 **Figure S1:** Inter day reproducibility of reporter peptide spiking. One serum specimen was measured three times on four different days. CP-AP mean value: 31.9 μmol/L. SD: 3.3. CV: 10.2%. The central box represents the values from the lower to upper quartile (25 to 75 percentile). The middle line represents the median. The horizontal line extends from the minimum to the maximum value. Click here for file
