# Supplementary figures and images for: Functional protease profiling with reporter peptides in serum specimens of colorectal cancer patients: demonstration of its routine diagnostic applicability
Source: J Exp Clin Cancer Res. 2012 Jun 8;31(1):56. doi: 10.1186/1756-9966-31-56 (PMC3780806; doi:10.1186/1756-9966-31-56)

## Slide 1
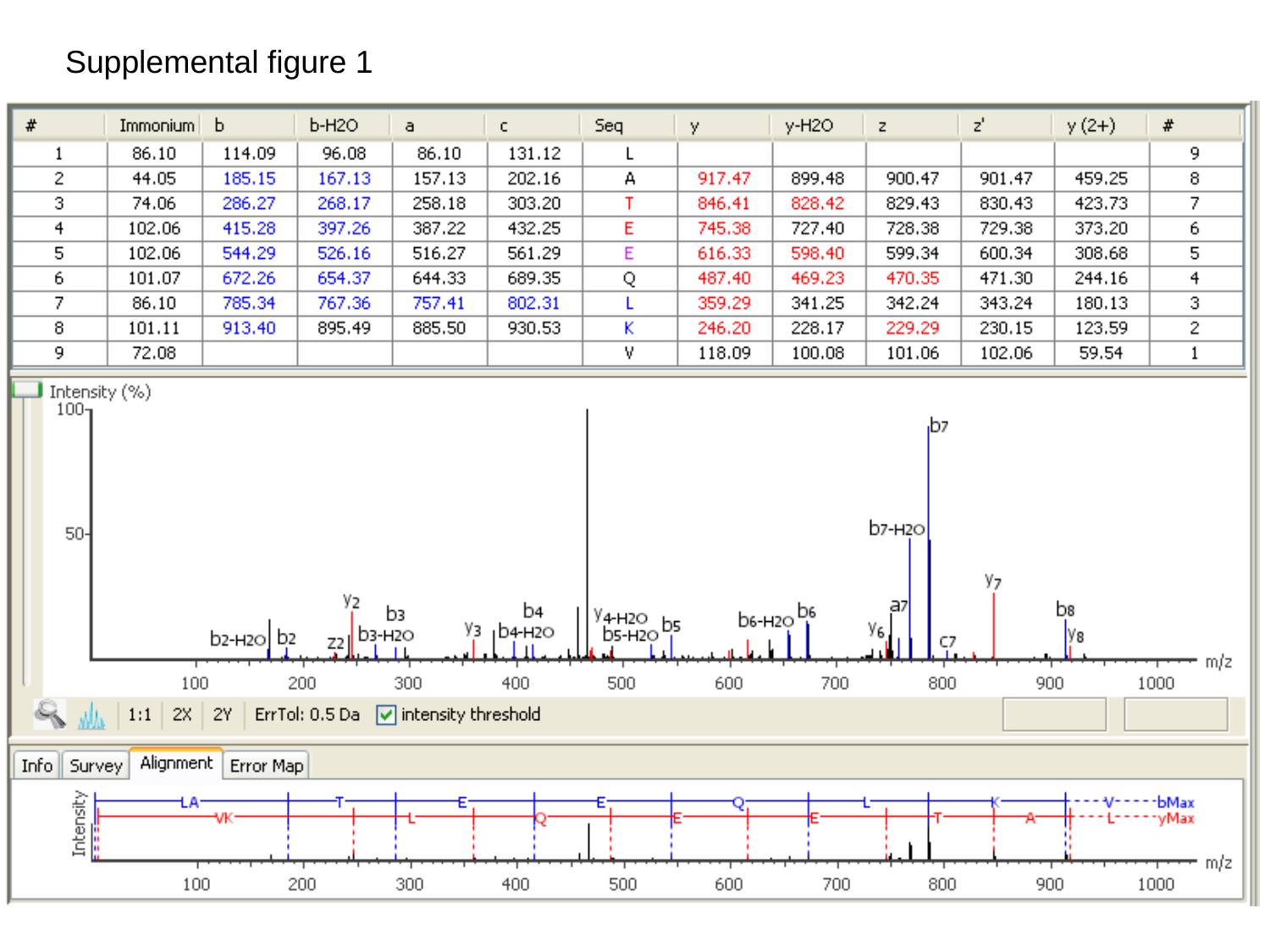

Supplemental figure 1

Supplement: Additional file 1 — Figure S1: Amino acid sequence confirmation of the anchor peptide Ahx-ateeqlkv (see Table 1). Print screen of the MS/MS spectra decoding of m/z 515.795 that was performed with PEAKS software (Bioinformatics Solutions). The unusual amino acid Ahx cannot be handled by the software and instead is displayed as Lysine (L) that is an isomer of Ahx and thus produces a fragment with the same mass. [file 1756-9966-31-56-S1.ppt]

## Slide 1
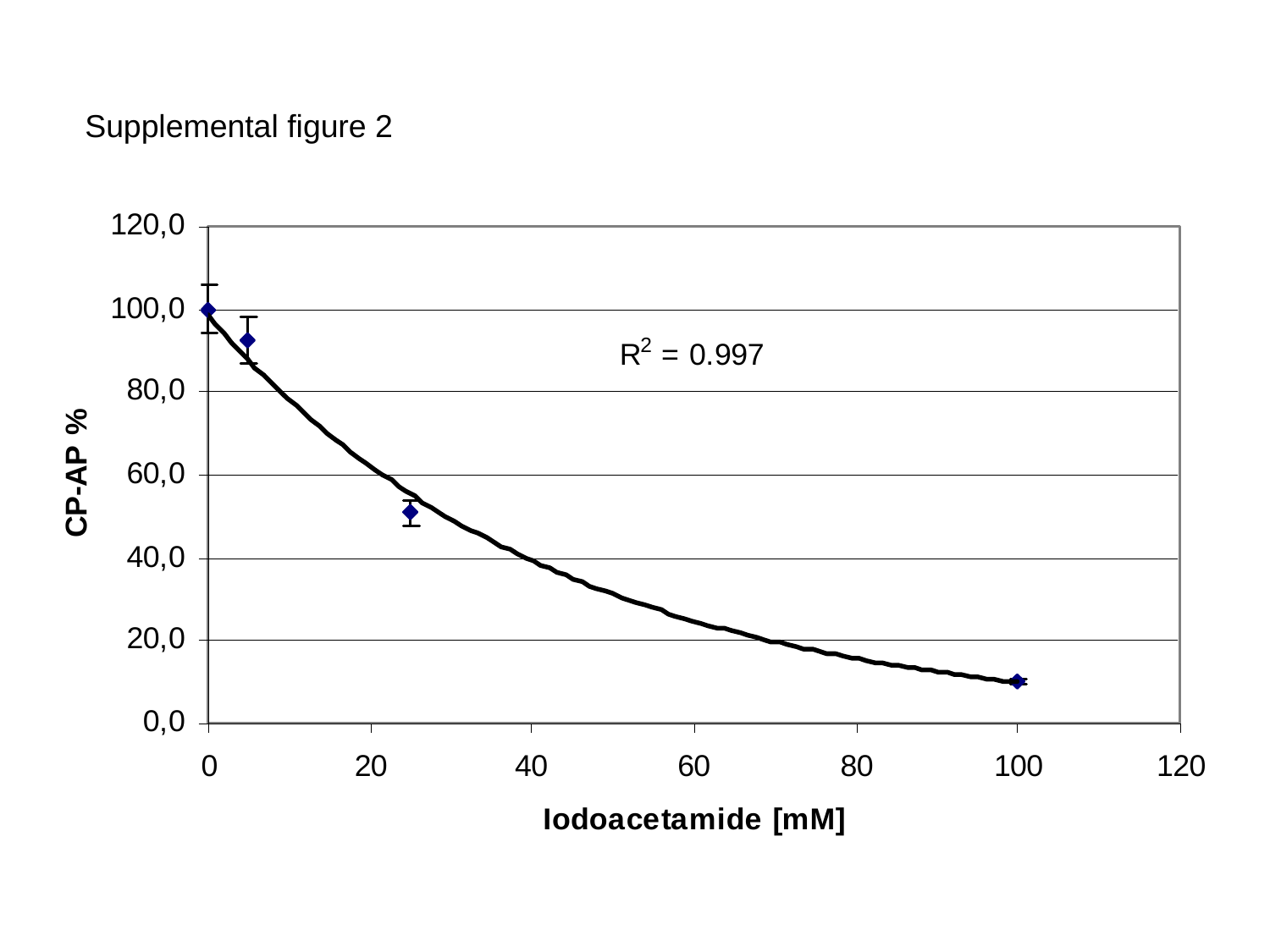

Supplemental figure 2

Supplement: Additional file 2 — Figure S2: Inhibition of protease-activity with iodoacetamide. The protease inhibitor iodoacetamide together with CP-RP and IS was added to a serum specimen from one tumor patient and incubated for 22 h prior to LC-MS analyis. Iodoacetamide concentrations ranged from 5 to 25 and 100 mmol/L. The CP-AP concentration of the serum specimen without iodoacetamide was set to 100%. Measurements were performed in triplicate and the squares indicate median values, error bars indicate standard deviations. The exponential regression was calculated with Excel (Microsoft) and the coefficient of determination (R2) is shown in the graph. [file 1756-9966-31-56-S2.ppt]

## Slide 1
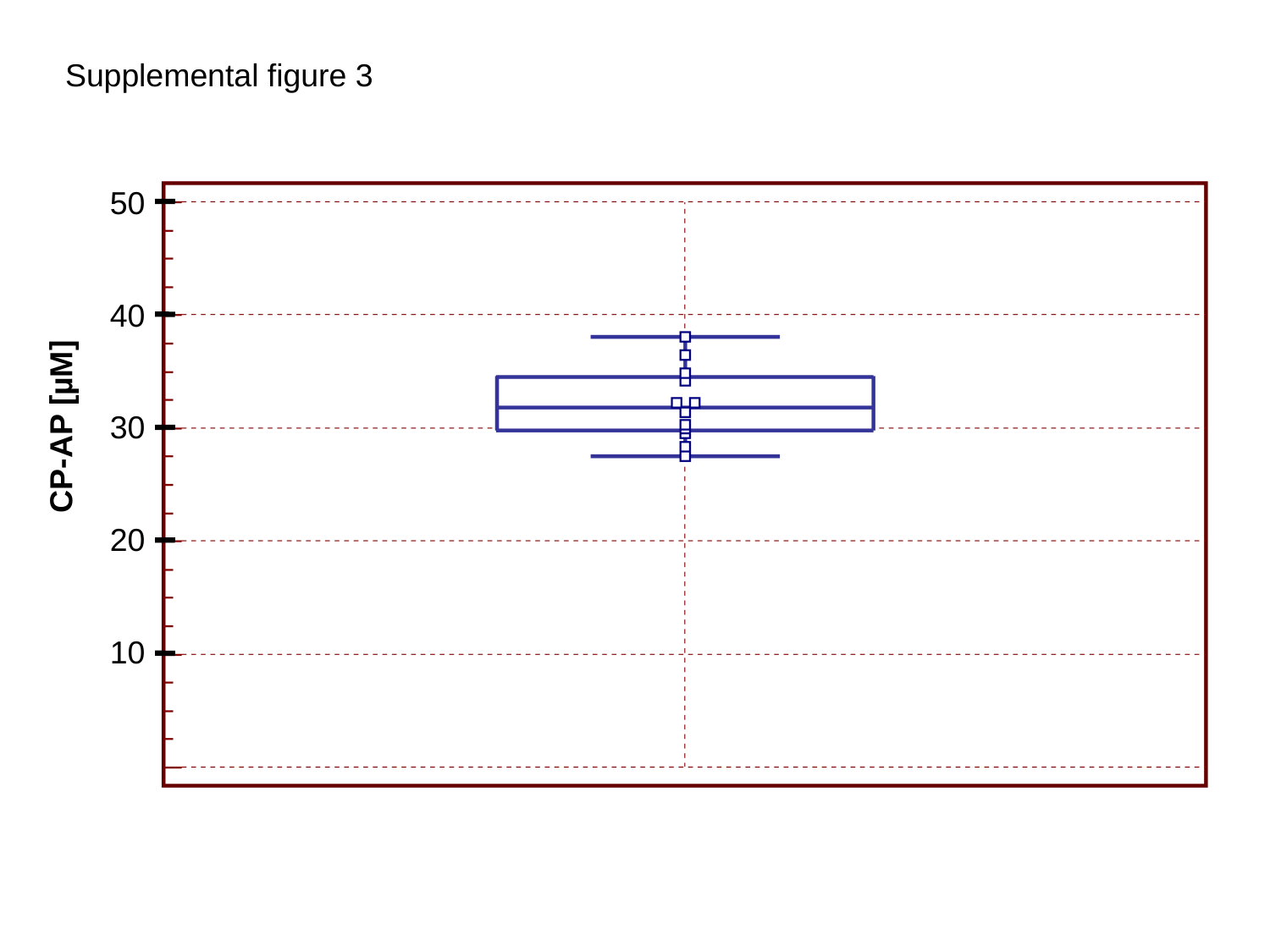

Supplemental figure 3
50
40
30
CP-AP [µM]
20
10

Supplement: Additional file 3 — Figure S1: Inter day reproducibility of reporter peptide spiking. One serum specimen was measured three times on four different days. CP-AP mean value: 31.9 μmol/L. SD: 3.3. CV: 10.2%. The central box represents the values from the lower to upper quartile (25 to 75 percentile). The middle line represents the median. The horizontal line extends from the minimum to the maximum value. [file 1756-9966-31-56-S3.ppt]
